# Knotless seton for perianal fistulas: feasibility and effect on perianal disease activity

**DOI:** 10.1038/s41598-020-73737-2

**Published:** 2020-10-07

**Authors:** Merel E. Stellingwerf, Michiel T. J. Bak, E. Joline de Groof, Christianne J. Buskens, Charlotte B. H. Molenaar, Krisztina B. Gecse, Willem Nerkens, Tim Horeman, Willem A. Bemelman

**Affiliations:** 1 Department of Surgery, Amsterdam UMC, University of Amsterdam, Post box 22660, 1100 DD Amsterdam, The Netherlands; 2 Proctos Kliniek, Bilthoven, The Netherlands; 3 Department of Gastroenterology and Hepatology, Amsterdam UMC, University of Amsterdam, Amsterdam, The Netherlands; 4 MediShield B.V., Delft, The Netherlands; 5 Delft University of Technology, Delft, The Netherlands

**Keywords:** Anal diseases, Inflammatory bowel disease, Crohn's disease

## Abstract

Patients with perianal fistulas are frequently treated by a knotted seton which is well-known for causing complaints. We aimed to assess the feasibility of the knotless SuperSeton and advantages with respect to perianal disease activity. In a prospective cohort study, we included all consecutive adult patients with a knotted seton in situ or a perianal fistula requiring new seton drainage. Primary endpoint was seton feasibility (maintenance of the connection for minimally three months). Secondary endpoints included improvement of the Perianal Disease Activity Index (PDAI), complications and re-interventions within three months of follow-up. PDAI scores of patients with a knotted seton were crossover compared to PDAI scores after knotless seton replacement. Sixty patients (42% male, mean age 42 (SD 13.15), 41 with Crohn’s disease) were included between August 2016 and April 2018. Of 79 knotless setons, 69 (87.3%) stayed connected for ≥ 3 months. Overall, the knotless seton significantly decreased discharge (P = 0.001), pain (P < 0.001) and induration (P < 0.001) measured by the PDAI when compared to baseline. In patients with a knotted seton, replacement by the knotless seton significantly decreased discharge (P = 0.005) and pain (P < 0.001) measured by the PDAI. Furthermore, 71% of patients reported fewer cleaning problems compared to the knotted seton. Ten patients developed a perianal abscess, and five patients required a re-intervention. This study supports the feasibility of the knotless seton with promising short-term results. The knotless seton might be preferred over the knotted seton in terms of perianal disease activity.

## Introduction

Perianal fistulas are associated with local pain, discharge, and considerable morbidity rates including sphincter and perineal tissue destruction causing disability^1,2^. The main cause of perianal fistula development is sepsis originating from the crypt glands. Other aetiologies include Crohn’s disease (CD), chronic fissures, malignancies, radiation, trauma or foreign bodies. The estimated incidence of perianal fistulas in Europe is 1.2–2.8 per 10,000 and in the Netherlands, 2850 patients require surgical intervention annually according to the Dutch National Medical Registration (Prismant 2001)^3^. Surgical therapy consists of lay open or fistulectomy for simple or low perianal fistulas (crossing the lower one-third of the external sphincter) resulting in high healing and low recurrence rates.

Unfortunately, a substantial number of patients present with a complex or high perianal fistula (crossing the upper two-third of the external sphincter), requiring a staged approach or chronic seton drainage. Regardless of the chosen strategy, the first step should always be placement of a surgical non-cutting seton for at least 4–6 weeks to maintain patency of the tract for optimal drainage and to prevent recurrent abscess formation^4,5^. In patients with Crohn’s perianal fistulas, adequate drainage is explicitly crucial before starting immunosuppressive treatment^6,7^. The ultimate treatment goal is fistula closure, however, long-term success rates remain moderate with less than 60% in most series, especially in CD patients with complex fistulas with multiple tracts^8,9^.

The seton was first described by Hippocrates, who used horse hair wrapped around a lint thread. Nowadays, vessel loops or sutures are mainly used as setons, depending on the surgeons preferences^10^. The ends of the seton drain are connected by a knot or by knotted suture threads. A knotted seton is relatively difficult to clean, and the knot has a tendency to migrate into the opening of the fistula tract which is very uncomfortable. To inventory complaints, a web-based survey was performed by the Dutch Crohn and Ulcerative Colitis Association (CCUVN). Twenty-four out of 46 patients (52%) reported to have daily complaints caused by the knot. Furthermore, 50% of the patients required re-interventions related to the knot. In order to improve these complaints and to avoid re-interventions, MediShield B.V. has developed a knotless seton, the SuperSeton. This new type of seton comes with a secure insert system that connects the two ends of the drain to form a smooth closed loop. Up till now one other knotless seton is available, the Comfort Drain (A.M.I., Feldkirch, Austria)^11^.

The aim of this study was to assess the feasibility of the knotless seton and advantages with respect to perianal disease activity in patients with cryptoglandular and Crohn’s perianal fistulas.

## Method

### Design and participants

In a prospective cohort study in the Amsterdam University Medical Center and the Proctos Kliniek (specialized referral clinic), consecutive patients ≥ 18 years of age with a new or recurrent Crohns or cryptoglandular perianal fistula, with either a knotted seton in situ or planned for new seton drainage, were included. Patients with a rectovaginal fistula or a defunctioning ostomy were excluded. Furthermore, patients with a life expectancy of less than two years, patients unable to read or understand the questionnaires or patients with dementia or altered mental status were excluded.

This study was conducted according to the principles of the declaration of Fortalezea Brazil (October 2013) and in accordance with the Medical Research Involving Human Subjects Act (WMO). Data management, monitoring and reporting of the study were carried out in accordance with the ICH GCP guidelines. Data was reported following the STROBE Checklist for cohort studies. Approval was obtained from the Medical Ethical Committee at the Amsterdam UMC in Amsterdam and the study was registered at ClinicalTrials.gov (Study ID: NCT03654482, date: 31/8/2018). Informed consent was obtained from all eligible patients.

### Primary and secondary endpoints

The primary endpoint was seton feasibility defined as maintenance of the connection of the knotless seton for minimally three months. Secondary endpoints were perianal disease activity as measured by the Perianal Disease Activity Index (PDAI)^12^, procedure time, complications and re-interventions within three months follow-up. The PDAI is the gold standard for evaluating the severity of perianal disease, and includes both patient reported outcome measurements and physical assessment by the treating specialist. The PDAI consists of five items: discharge, pain, restriction of sexual activity, type of perianal disease, and degree of induration. Each category is graded on a scale ranging from no symptoms (zero points) to severe symptoms (four points). The PDAI score was initially developed for the evaluation of Crohn’s perianal fistulas, however, as no such scoring system exists for cryptoglandular fistulas, the PDAI score was used for both fistula aetiologies in this study. The PDAI scores of patients with a knotted seton in situ at baseline were compared in a crossover fashion to the PDAI scores after replacement by the knotless seton at three months follow-up. Hence these patients served as their own controls. Additionally, all patients who ever had a knotted seton were asked to fill out a short questionnaire (Supplementary Fig. 1) before knotless seton insertion and three months thereafter to assess the failure rate (disconnection of the knot), cleaning problems and complaints (pain, itchiness, irritation and discharge) related to the knot. Cleaning problems and complaints were evaluated by a VAS scale ranging from 0 (no complaints) to 10 (severe complaints).

### The knotless seton and surgical procedure

**Figure 1:**
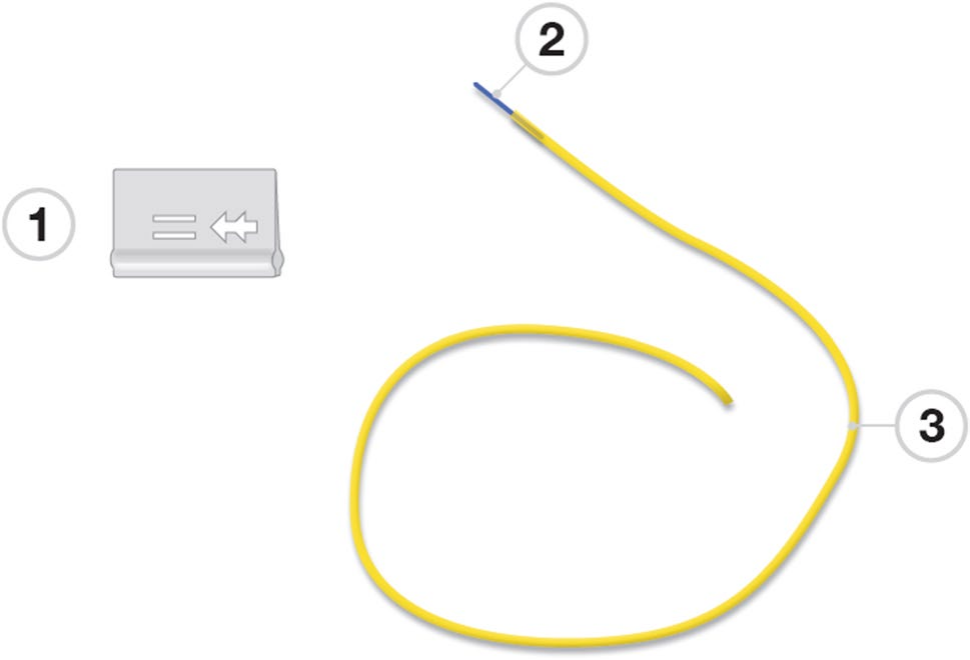
1 applier, 2 insert, 3 drainage tube.

**Figure 2:**
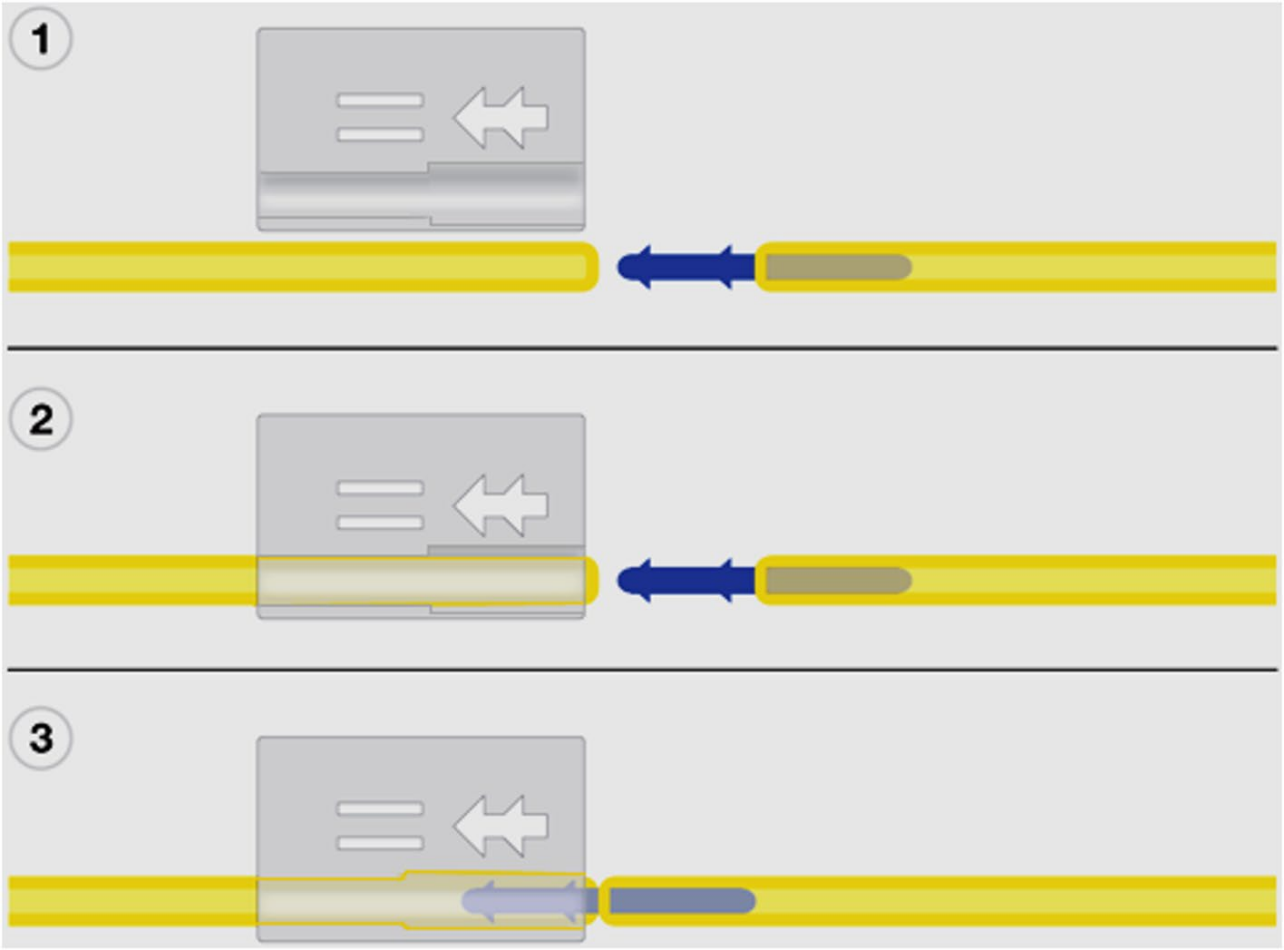
Usage of the applier to form a smooth connection.

The applier of the SuperSeton (MediShield B.V., Delft, The Netherlands) is made of medical grade 3D print material, the insert of medical grade polypropylene, and the tube of medical grade silicone (Fig. 1). The setons including the insert and applier are delivered sterile (Fig. 2). The insert has two arrow shaped secure hooks assuring a safe connection. Placement of the knotless seton was performed by nine experienced colorectal surgeons (five at the Amsterdam UMC and four at the Proctos Kliniek). All surgeons had to practice the knotless seton placement three times in vitro under supervision of a MediShield B.V. associate. Thereafter, patients with a conventional knotted seton in situ were seen at the outpatient clinic were the knotted seton was replaced by the knotless seton. In case patients did not have a conventional knotted seton in situ or in patients with considerably complicated perianal disease, the knotless setons were placed at the operating theatre in day care setting.

### Sample size and statistics

The sample size calculation is based on a clinically relevant decrease in the PDAI. The PDAI has five items with a total score ranging from 0 to 20, and a score of > 7 is considered as severe perianal disease. The CCUVN survey indicated that the PDAI reflected severe complaints in Crohn’s fistula patients, with high scores on all five scales (average 9.2). A decrease to 6.9 is considered to be clinically relevant (< 7 considered as moderate perianal complaints). The sample size needed to demonstrate superiority of the knotless seton with a significant decrease in PDAI compared to baseline is 60 patients (alpha 0.05, power 90%, and 5% drop-out). The proposed treatment is considered feasible if at least 70% of knotless setons stay in place for at least three months.

All data were collected in an electronic database by the study coordinator. Descriptive statistics were used to describe patient characteristics and outcomes. Comparison of continuous data between subgroups was performed by the unpaired t-test. The (preoperative) baseline PDAI scores were compared to (postoperative) PDAI scores within the same patient at three months follow-up. Results were analysed using the paired t-test for numerical data and the McNemar’s test for categorical data. A two tailed p-value of < 0.05 was considered statistically significant. Statistical analysis was performed using IBM SPSS for Windows version 24 (IBM Corp., Armonk, NY, United States).

### Conference presentation

This paper has been presented during the Alpine Colorectal Meeting (January 2017), the European Crohn and Colitis Organisation (February 2017), and the NVGE Digestive Disease Days (March 2017).

## Results

### Patient and treatment characteristics

**Figure 3:**
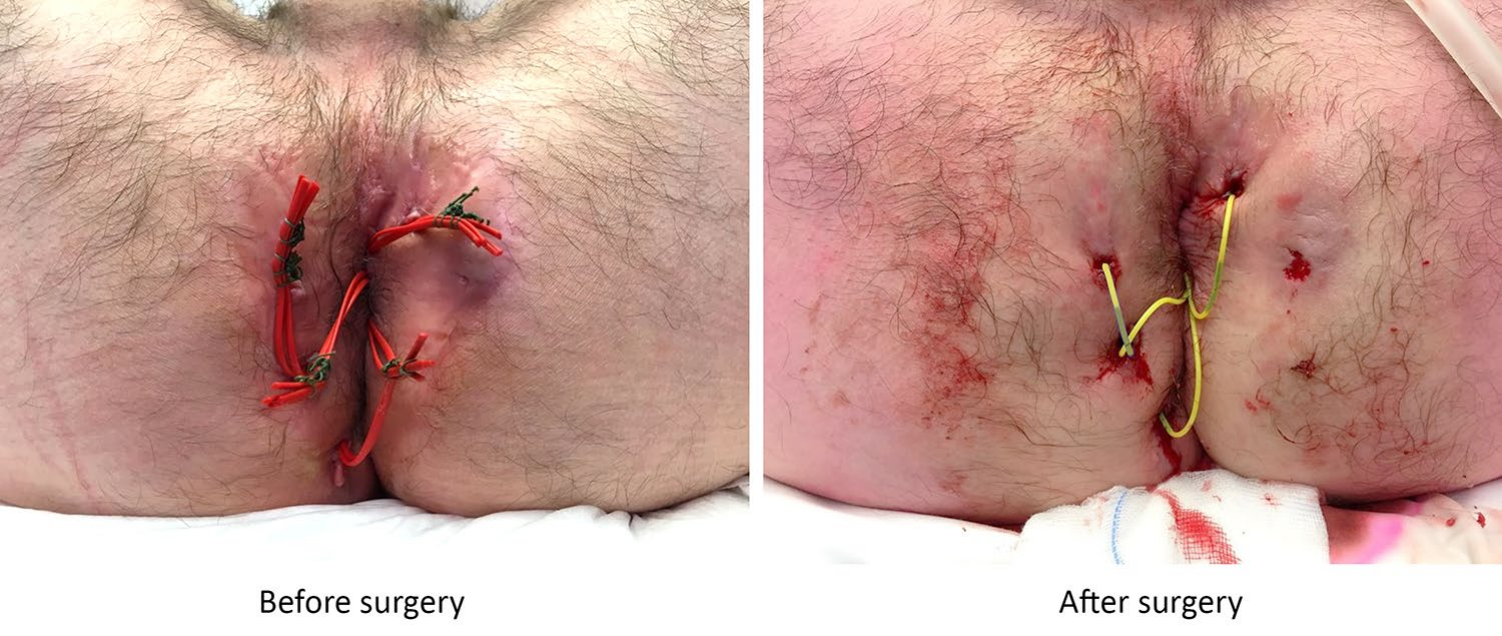
Knotted setons replaced by SuperSetons.

In total, 60 patients were included between August 2016 and April 2018, 41 (68.3%) had underlying CD and 19 (31.7%) had cryptoglandular fistulas. The mean age at inclusion was 42.0 years (SD 13.15) and 58.3% were female (Table 1). In total, 79 knotless setons were placed with a median of one (range 1–3) seton per patient. At baseline, 37 patients (61.7%) had one or more conventional knotted setons in situ (vessel loops in 29 and sutures in eight) for median five months (range 0.5–216). Twenty-nine patients received knotless setons at the outpatient clinic, and eight patients, who had complicated perianal disease, had their seton(s) exchanged in theatre (Fig. 3). Twenty-three patients had perianal fistulas with no seton in situ and a knotless seton was placed in the operating theatre in day care setting with a median procedure time of 21 min (range 11–60). Thirty-four (82.9%) of the Crohn’s disease patients received medical treatment at baseline. During follow-up one patient stopped with biological treatment (self-initiated), one patient switched from adalimumab to infliximab and one patient stopped with methotrexate.

**Table 1:** Baseline characteristics.

	N = 60
**Female (n, %)**	35 (58.3)
**Age in years (mean, SD)**	42.0 (13.15)
**Recurrent fistula (n, %)**	48 (80.0)
**Crohn’s disease (n, %)**	41 (68.3)
Medication	34 (82.9)
5-ASA	1 (2.4)
Steroids	4 (9.8)
Immunomodulators	18 (43.9)
Biologicals	24 (58.5)
**Knotted seton(s) in situ (n, %)**	37 (61.7)
Vessel loop	29 (78.4)
Suture	8 (21.6)
**Ever treated by knotted seton(s) (n, %)**	56 (93.3)
Loosening of connection	26 (46.4)
Complaints of knot	46 (82.1)
Discharge	21 (37.5)
Pain	34 (60.7)
Itchiness	16 (28.6)
Irritation	33 (58.9)

### Outcome parameters and follow-up

At three months follow-up, 69 out of 79 (87.3%) of the knotless setons stayed connected. The mean total PDAI score of the total study group significantly decreased from 9.55 (SD 3.73) to 7.13 (SD 3.66) (P < 0.001) at three months follow-up. Looking at each of the five subscales of the PDAI, a significant decrease was seen in discharge (P = 0.001), pain (P < 0.001) and induration (P < 0.001) (Table 2). In patients with cryptoglandular or CD fistulas with a knotted seton in situ at baseline (n = 37), the mean total PDAI score significantly decreased from 9.16 (SD 3.59) to 6.76 (SD 3.48) after replacement by the knotless seton (P < 0.001). In these patients, a significant decrease was seen in the subscales of discharge (P = 0.005) and pain (P < 0.001). In patients with cryptoglandular or CD fistulas with a newly placed knotless seton for an undrained perianal fistula (n = 23), the mean total PDAI score significantly decreased from 10.17 (SD 3.95) to 7.74 (SD 3.94) (P = 0.001). In these patients, only the subscale of induration significantly decreased (P < 0.001).

**Table 2:** Mean PDAI scores at baseline and three months follow-up.

	PDAI at baseline	PDAI at 3 months	P-value
**Total PDAI (n = 60; mean, SD)**	9.55 (3.73)	7.13 (3.66)	< 0.001
PDAI discharge	2.22 (1.12)	1.72 (1.21)	0.001
PDAI pain	2.05 (1.28)	1.67 (0.99)	< 0.001
PDAI sexual activity	1.89 (1.68)	1.57 (1.60)	0.068
PDAI type perianal disease	2.27 (0.69)	2.23 (0.72)	0.419
PDAI induration	1.60 (1.25)	0.92 (1.24)	< 0.001
**PDAI Crohn + knotted in situ (n = 24; mean, SD)**	9.58 (3.69)	6.67 (3.00)	< 0.001
PDAI discharge	2.29 (1.04)	1.50 (1.29)	0.005
PDAI pain	2.42 (1.38)	1.17 (0.82)	< 0.001
PDAI sexual activity	2.25 (1.89)	1.65 (1.84)	0.030
PDAI type perianal disease	2.33 (0.70)	2.29 (0.69)	0.328
PDAI induration	1.21 (1.32)	0.96 (1.27)	0.408
**PDAI Crohn + undrained (n = 17; mean, SD)**	10.71 (3.87)	8.41 (4.23)	0.007
PDAI discharge	2.29 (1.16)	2.06 (1.09)	0.260
PDAI pain	1.88 (1.27)	1.53 (1.38)	0.269
PDAI sexual activity	2.19 (1.72)	1.75 (1.65)	0.276
PDAI type perianal disease	2.41 (0.80)	2.41 (0.80)	
PDAI induration	2.35 (1.12)	1.06 (1.30)	< 0.001
**PDAI cryptoglandular + knotted (n = 13; mean, SD)**	8.39 (3.38)	6.92 (4.35)	0.158
PDAI discharge	2.00 (1.23)	1.69 (1.32)	0.416
PDAI pain	1.92 (1.19)	0.92 (0.76)	0.001
PDAI sexual activity	1.33 (1.30)	1.42 (1.38)	0.754
PDAI type perianal disease	2.00 (0.00)	1.84 (0.56)	0.337
PDAI induration	1.31 (0.86)	1.08 (1.32)	0.570
**PDAI cryptoglandular + undrained (n = 6; mean, SD)**	8.67 (4.13)	5.83 (2.32)	0.059
PDAI discharge	2.17 (1.17)	1.67 (1.03)	0.363
PDAI pain	1.33 (0.82)	0.67 (0.52)	0.235
PDAI sexual activity	1.00 (1.10)	1.17 (1.17)	0.741
PDAI type perianal disease	2.17 (0.98)	2.33 (0.82)	0.363
PDAI induration	1.67 (1.37)	0.00 (0.00)	0.031

#### Crohn’s perianal fistulas

**Figure 4:**
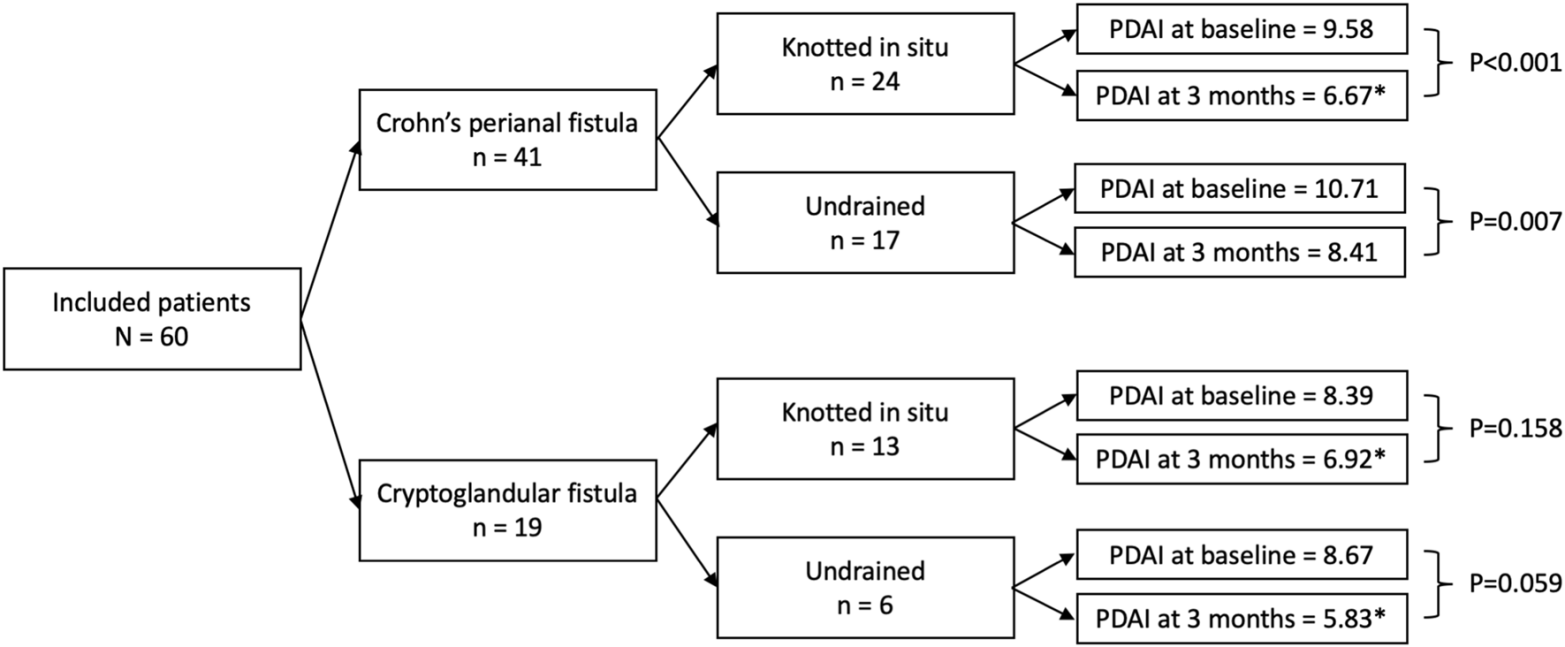
Flowchart of subgroups and total PDAI. *Clinically relevant decrease in PDAI.

In patients with CD and a knotted seton in situ at baseline (n = 24) the mean total PDAI score significantly decreased from 9.58 (SD 3.69) to 6.67 (SD 3.00)(P < 0.001) after knotless seton replacement (Fig. 4). In this patient group the subscales of discharge (P = 0.005), pain (P < 0.001) and restriction of sexual activity (P = 0.030) significantly decreased. In CD patients with a newly placed knotless seton for an undrained perianal fistula or abscess (n = 17) the mean total PDAI score also significantly decreased from 10.71 (SD 3.87) to 8.41 (SD 4.23)(P = 0.007), however, only the subscale of induration showed a significant decrease (P < 0.001).

#### Cryptoglandular perianal fistulas

In patients with a cryptoglandular fistula and a knotted seton in situ (n = 13) the mean total PDAI score did not significantly change after replacement with the knotless seton (8.39 (SD 3.38) to 6.92 (SD 4.35); P = 0.158) (Fig. 4). Only the subscale reporting on pain significantly decreased from 1.92 (SD 1.19) to 0.92 (SD 0.76) (P = 0.001). In patients with a newly placed knotless seton for an undrained cryptoglandular fistula (n = 6) also no change in the mean total PDAI score was seen after surgical placement of the knotless seton [8.67 (SD 4.13) to 5.83 (SD 2.32); P = 0.059]. Similar to undrained Crohn’s perianal fistulas, only the subscale of induration significantly decreased (P = 0.031).

#### Complaints related to the knot

Of all 60 patients, 56 (93.3%) had had at least one conventional knotted seton (the total number of knotted seton treatments in these patients was 135). Disconnection of the knot had occurred in 47 out of 135 (34.8%) setons, of which 42 of the setons were replaced in theatre. Forty-six of the 56 patients (82.1%) experienced complaints specifically related to the knot with severity graded as median 8 (range 1–10) on a scale of 0 to 10. Bicycling, walking and sitting were most often mentioned as activities that aggravated symptoms. Forty out of the 56 patients (71.4%) reported fewer problems with cleaning after defecation with the knotless seton when compared to the previous knotted seton. Patients graded the severity of the overall cleaning problems on a scale of 0 to 10 as a mean of 6.30 (SD 2.89) with the knotted seton and this significantly decreased to a mean of 3.48 (SD 3.10) with the knotless seton (P < 0.001). The decrease in cleaning problems was mainly due to less pain (P = 0.008), irritation (P < 0.001), and discharge of blood, pus and faeces through the fistula (P = 0.004) with the knotless seton.

### Complications

No complications occurred during placement of the knotless setons at the outpatient clinic and operating theatre. Postoperatively, five patients reported newly developed or increased perianal pain complaints possibly related to the knotless seton; in two patients the insert system slightly detached but no re-intervention was indicated, in one patient the fistula tract was not adequately drained by the knotless seton with an inflammatory mass on MRI (without any evidence of abscess formation) for which ciprofloxacin was started, in one patient the knotless seton was removed due to perianal pain complaints but during follow-up this seemed to be caused by a de novo bladder carcinoma, and in one patient no clear cause was found for the perianal pain complaints (Table 3). Furthermore, 10 patients developed a perianal abscess during 3 months follow-up (in six patients due to inadequate drainage of the knotless seton), for which a surgical re-intervention was performed in two of the patients. Another patient underwent a re-intervention for a longer existing painful fissure which was closed surgically. Lastly, surgical re-intervention was also required in two of the nine patients who lost the knotless seton. In total five patients (8.3%) underwent a surgical re-intervention during the follow-up of the study.

**Table 3:** Postoperative complications.

Complications	Crohn’s		Cryptoglandular	
	Knotted seton exchange (n = 24)	New seton placement (n = 17)	Knotted seton exchange (n = 13)	New seton placement (n = 6)
**Increased perianal pain (n, %)**				
Detached insert system	2 (8.3)			
Inadequate drainage	1 (4.2)			
Other reason/ unknown	1 (4.2)	1 (5.9)		
**Perianal abscess (n, %)**				
Inadequate drainage	4 (16.7)	1 (5.9)	1 (7.7)	
New location	2 (8.3)	2 (11.8)		
**Surgical re-intervention (n, %)**				
Abscess	2 (8.3)			
Painful fissure		1 (5.9)		
Loosening of connection		2 (11.8)		

## Discussion

The knotless SuperSeton is a feasible technique for patients with new and recurrent cryptoglandular or Crohn’s perianal fistulas as 69 out of 79 setons (87.3%) stayed connected for at least three months. Insertion of the knotless seton significantly improved the perianal disease activity as measured by the PDAI, with a decrease in discharge, pain and induration. Also, after replacement of the knotted seton, a significant decrease in the PDAI was seen. Subgroup analysis of the two underlying aetiologies showed that the decrease in PDAI remained significant for Crohn’s perianal fistulas but not for cryptoglandular fistulas. Knotless seton (re)placement appeared clinically relevant in all patients with a conventional knotted seton in situ, but not in Crohn’s patients with undrained fistulas.

The study of Kristo et al.^13^ reported similar results with significantly improved physical and mental health and less burning sensation and pruritis in 22 patients treated with the Comfort Drain, the only other knotless seton which is commercially available. In our subjective experience, after implementing the Comfort Drain in daily clinical practice, this knotless seton appeared to fall out easily and the extra arrow shaped secure hooks in the insert system of the novel knotless seton aim to prevent this. Another advantage is the applier that comes with the knotless seton. The applier provides stability and prevents buckling during placement making the procedure less difficult for the surgeon probably resulting in shorter operation times (median 21 min in our study versus 26 min in the study of Kristo et al. ^13^). Furthermore, the knotless seton has a softer insert which is easier to bend, potentially improving patient comfort during sitting and physical activities.

Differentiating Crohn’s from cryptoglandular perianal fistulas is essential in evaluating the outcomes of knotless seton treatment as Crohn’s fistulas have a different aetiology and are more often refractory to surgery. In our study, a significant decrease in the PDAI score was seen in perianal fistulas of Crohn’s origin as opposed to cryptoglandular fistulas. This might be due to differences in pathogenesis or experience of symptoms, however, the study was not powered to evaluate Crohn’s and cryptoglandular fistulas separately. Also, the higher complication rate in Crohn’s fistulas compared to cryptoglandular fistulas (34.1% versus 5.3% respectively) with all five re-interventions performed in Crohn’s fistulas warrants consideration. The diameter of the knotless seton as used in this study might be too thin to adequately drain complex Crohn’s fistulas with a large diameter and drainage by a thicker (or double) version might be more appropriate for this patient group. On the other hand, previous data also showed higher complication rates in CD patients treated by the conventional knotted seton (23.5%) compared to non-CD patients (8.4%)^14^. Furthermore, it is likely that the patients in our study who were being treated at two expert centres, had more complex perianal fistulas with a higher chance of postoperative complications than patients treated elsewhere. Our complication rates should therefore be interpreted with caution.

The clinical relevance of statistically significant differences in the total PDAI score remains disputable, however, a total score of > 7 is considered as serious perianal disease and therefore a decrease to 6.9 is considered to be clinically relevant. In our study, such a decrease in PDAI scores was seen in both Crohn’s patients and cryptoglandular fistula patients with a knotted seton in situ and in cryptoglandular fistula patients with an undrained fistula at baseline. In undrained fistulas and abscesses a decrease in PDAI score after placement of a new seton is likely to be due to the decrease in inflammation, confounding the effect of the knotless connection itself. Therefore, relatively larger decreases in PDAI might be expected compared to patients with a knotted seton in situ, which was however not seen in our results (Fig. 4). In patients with a knotted seton in situ and a decrease in the PDAI score after replacement by the knotless seton, the decrease can be attributed to the difference between setons including the knotless connection. Also, replacement of the knotted seton by the knotless seton is suggested to be clinically relevant in both Crohn’s and cryptoglandular fistula patients following the results of our subgroup analysis. Physicians might therefore consider to replace knotted setons for comfort purposes if initial drainage is achieved and chronic drainage is necessary in the long-run. For temporary drainage of a fistula it might not be worthwhile to replace a knotted seton keeping in mind patient burden and associated costs (54 dollar for a knotless seton versus 8 dollar for a conventional knotted seton).

There are some limitations to our study. As previously mentioned, no definite conclusions can be drawn from the subgroup analyses due to small numbers. Also, the nature of our study design did not allow for a direct comparison between the conventional knotted seton and the knotless seton in terms of their ability to stay connected. Patients previously treated by a conventional knotted seton reported in a non-validated retrospective questionnaire that 34.8% had lost its connection after an unknown period of time. Treatment by the knotless seton resulted in disconnection in 12.7% of the patients within short-term follow-up of three months, therefore no conclusions can be drawn from differences between these percentages. A comparison was made between the conventional knotted seton and the knotless seton in terms of PDAI scores in a crossover fashion, which demonstrates clinically relevant superiority compared to the conventional knotted seton. However, it should be borne in mind that this might be confounded by the natural course of improvement of the PDAI during drainage over time. Lastly, in this pragmatic study, in some of the patients double vessel loops were used with subsequent larger knots which might have influenced the results.

The results of this study support the feasibility of the knotless seton with promising short-term results. Insertion of the knotless seton significantly improved the perianal disease activity, with a decrease in discharge, pain and induration. Also, replacement of the knotted seton by the knotless seton was clinically relevant for both patients with Crohn’s and cryptoglandular fistulas. Therefore, the knotless seton might be preferred over the conventional knotted seton in patients with perianal fistulas, especially if the conventional seton needs to be replaced. This is an intervention that can easily be done at the outpatient department. As our single-arm study design did not allow for direct comparison, future studies should aim to compare the knotless seton with different knotted techniques used in practice.

## Supplementary information

Supplementary file1

Supplementary file2

## References

[1] Vogel JD, Johnson EK, Morris AM, Paquette IM, Saclarides TJ, Feingold DL (2016). Clinical practice guideline for the management of anorectal abscess, fistula-in-ano, and rectovaginal fistula. Dis. Colon Rectum.

[2] Vigano C, Losco A, Caprioli F, Basilisco G (2011). Incidence and clinical outcomes of intersphincteric abscesses diagnosed by anal ultrasonography in patients with crohn's disease. Inflamm. Bowel Dis..

[3] Zanotti C, Martinez-Puente C, Pascual I, Pascual M, Herreros D, Garcia-Olmo D (2007). An assessment of the incidence of fistula-in-ano in four countries of the European Union. Int. J. Colorectal Dis..

[4] Van Assche G, Dignass A, Reinisch W, van der Woude CJ, Sturm A, De Vos M (2010). The second European evidence-based Consensus on the diagnosis and management of Crohn's disease: special situations. J. Crohn Colitis..

[5] Nederlandse vereniging voor Heelkunde. *Richtlijn Proctologie*. https://richtlijnendatabase.nl.

[6] Regueiro M, Mardini H (2003). Treatment of perianal fistulizing Crohn's disease with infliximab alone or as an adjunct to exam under anesthesia with seton placement. Inflamm. Bowel Dis..

[7] Hyder SA, Travis SP, Jewell DP, Mc CMNJ, George BD (2006). Fistulating anal Crohn's disease: results of combined surgical and infliximab treatment. Dis. Colon Rectum.

[8] Lee MJ, Parker CE, Taylor SR, Guizzetti L, Feagan BG, Lobo AJ (2018). Efficacy of medical therapies for fistulizing crohn's disease: systematic review and meta-analysis. Clin. Gastroenterol. Hepatol..

[9] Stellingwerf ME, van Praag EM, Tozer PJ, Bemelman WA, Buskens CJ (2019). Systematic review and meta-analysis of endorectal advancement flap and ligation of the intersphincteric fistula tract for cryptoglandular and crohn's high perianal fistulas. BJS Open..

[10] Subhas G, Singh Bhullar J, Al-Omari A, Unawane A, Mittal VK, Pearlman R (2012). Setons in the treatment of anal fistula: review of variations in materials and techniques. Dig. Surg..

[11] Riss SBHT, Stift A (2014). The comfort drain: a new device for treating complex anal fistula. Tech. Coloproctol..

[12] Irvine EJ (1995). Usual therapy improves perianal Crohn's disease as measured by a new disease activity index. McMaster IBD Study Group. J. Clin. Gastroenterol..

[13] Kristo I, Stift A, Staud C, Kainz A, Bachleitner-Hofmann T, Chitsabesan P (2016). The type of loose seton for complex anal fistula is essential to improve perianal comfort and quality of life. Colorectal Dis..

[14] Galis-Rozen E, Tulchinsky H, Rosen A, Eldar S, Rabau M, Stepanski A (2010). Long-term outcome of loose seton for complex anal fistula: a two-centre study of patients with and without Crohn's disease. Colorectal Dis..

