# Author Correction: Knotless seton for perianal fistulas: feasibility and effect on perianal disease activity

**DOI:** 10.1038/s41598-021-88353-x

**Published:** 2021-04-19

**Authors:** Merel E. Stellingwerf, Michiel T. J. Bak, E. Joline de Groof, Christianne J. Buskens, Charlotte B. H. Molenaar, Krisztina B. Gecse, Willem Nerkens, Tim Horeman, Willem A. Bemelman

**Affiliations:** 1 Department of Surgery, Amsterdam UMC, University of Amsterdam, Post box 22660, 1100 DD Amsterdam, The Netherlands; 2 Proctos Kliniek, Bilthoven, The Netherlands; 3 Department of Gastroenterology and Hepatology, Amsterdam UMC, University of Amsterdam, Amsterdam, The Netherlands; 4 MediShield B.V., Delft, The Netherlands; 5 Delft University of Technology, Delft, The Netherlands

Correction to: *Scientific Reports* 10.1038/s41598-020-73737-2, published online 07 October 2020

In the original version of this Article, Merel E. Stellingwerf was incorrectly listed as the corresponding author. The correct corresponding author for this Article is Tim Horeman. Correspondence and request for materials should be addressed to t.horeman@tudelft.nl

This error has now been corrected in the HTML and PDF versions of the Article, and in the Supplementary Information file 1 that accompanies the Article.

