# Supplementary material for: Knotless seton for perianal fistulas: feasibility and effect on perianal disease activity
Source: Sci Rep. 2020 Oct 7;10:16693. doi: 10.1038/s41598-020-73737-2 (PMC7541651; doi:10.1038/s41598-020-73737-2)
Supplement: Supplementary file 1 — Supplementary file1 [file 41598_2020_73737_MOESM1_ESM.docx]

**Knotless seton for perianal fistulas: feasibility and effect on perianal disease activity**
*Short title: Knotless seton for perianal fistulas*
Merel E. Stellingwerf BSc^1^, Michiel T.J. Bak BSc^2^, E. Joline de Groof MD, PhD^1^, Christianne J. Buskens MD, PhD^1^, Charlotte B.H. Molenaar MD^2^, Krisztina B. Gecse MD, PhD^3^, Willem Nerkens MSc^4^, Tim Horeman MSc, PhD^4,5^, Willem A. Bemelman MD, PhD^1^

^1^ Department of Surgery, Amsterdam UMC, University of Amsterdam, Amsterdam, The Netherlands
^2^ Proctos Kliniek, Bilthoven, The Netherlands

^3^ Department of Gastroenterology and Hepatology, Amsterdam UMC, University of Amsterdam, Amsterdam, The Netherlands

^4^ MediShield B.V., Delft, The Netherlands

^5^ Delft University of Technology, Delft, The Netherlands

**Corresponding author and requests for reprints:** Tim Horeman
 Delft University of Technology
 Mekelweg 5
 2628 CD Delft, Zuid-Holland
 The Netherlands
 Phone: +31 (0) 15 2784717

 E-mail: t.horeman@tudelft.nl

**Word count:** text 3145, abstract 248

**Conflicts of Interest and Source of Support:** W.A. Bemelman received a funding from Abbvie. W. Nerkens and T. Horeman are founders of MediShield B.V. which makes the knotless seton tested in this study. W.A. Bemelman received knotless seton kits free of charge from MediShield B.V. For the remaining authors none were declared. **Keywords:** Perianal Fistula; Perianal Crohn’s Disease; Knotless seton; Perianal Disease Activity Index
*This paper has been presented during the Alpine Colorectal Meeting (January 2017), the European Crohn and Colitis Organisation (February 2017), and the NVGE Digestive Disease Days (March 2017)*.
