# Supplementary material for: Knotless seton for perianal fistulas: feasibility and effect on perianal disease activity
Source: Sci Rep. 2020 Oct 7;10:16693. doi: 10.1038/s41598-020-73737-2 (PMC7541651; doi:10.1038/s41598-020-73737-2)
Supplement: Supplementary file 2 — Supplementary file2 [file 41598_2020_73737_MOESM2_ESM.docx]

**Supplementary Figure 1 – Questionnaire to assess complaints related to the knot**This questionnaire has been designed for this study to assess which complaints are specifically related to the knot in the seton used to drain your perianal fistula.

Please indicate which answers best describe your situation by placing a cross in one of the boxes: ⊗

**1) How many knotted setons have you been treated with in total?**

- 0
- 1
- 2
- 3
- More, namely ……….

**2) How many knotted setons do you currently have?**

- 0
- 1
- 2
- 3
- More, namely ……….

**3) For how long have you been treated with your current knotted seton(s)? (If you have multiple knotted setons, please answer this question for the knotted seton you have the longest)**

- 1-4 weeks
- 1-3 months
- 3-6 months
- 6-9 months
- 9-12 months
- More, namely ……….

**4) How often did you lose a knotted seton?**

- 0
- 1
- 2
- 3
- More, namely ……….

**5) How often has/have your knotted seton(s) been replaced in the operation room?**

- 0
- 1
- 2
- 3
- More, namely ……….

**6) Do you currently experience complaints when cleaning after defecating? If yes, which complaints? (multiple options are possible)**

- No
- Yes:
  - Pain
  - Itchiness
  - Irritation
  - Discharge of blood, pus or faeces through the fistula
  - Other, namely……………………………………………………………………………

**7) How bothersome are these complaints for you? Please rate the severity of the complaints.**

0 1 2 3 4 5 6 7 8 9 10

Not bothersome Really bothersome
 **8) Do you currently experience complaints specifically related to the knot?**

- No
- Yes:
  - Pain
  - Itchiness
  - Irritation
  - Discharge of blood, pus or faeces through the fistula
  - Other, namely……………………………………………………………………………

**9) How bothersome are these complaints for you? Please rate the severity of the complaints.**

0 1 2 3 4 5 6 7 8 9 10

Not bothersome Really bothersome

**10) Which activities increase your complaints related to the knot?**
(i.e. walking/ sitting/ standing/ bicycling)

…………………………………………………………………………………………………………………………………

**11) Which activities decrease your complaints related to the knot?**
(i.e. walking/ sitting/ standing/ bicycling)

…………………………………………………………………………………………………………………………………
